# SIRT1 Expression Is Associated with the Chemotherapy Response and Prognosis of Patients with Advanced NSCLC

**DOI:** 10.1371/journal.pone.0079162

**Published:** 2013-11-05

**Authors:** Tao Zhang, Ningning Rong, Juan Chen, Chengwei Zou, Haiyan Jing, Xiaolong Zhu, Wenlong Zhang

**Affiliations:** 1 Department of Cardiovascular Surgery, Provincial Hospital Affiliated to Shandong University, Shandong University, Jinan, Shandong Province, China; 2 Intensive Care Unit, Shandong provincial Chest Hospital, Jinan, Shandong Province, China; 3 Department of cardiothoracic surgery, Jinan seventh people's hospital, Jinan, Shandong Province, China; 4 Department of Pathology, Provincial Hospital Affiliated to Shandong University, Shandong University, Jinan, Shandong Province, China; Vanderbilt University Medical Center, United States of America

## Abstract

**Aim:**

The role of Sirtuin 1 (SIRT 1) in carcinogenesis is controversial. This study was to explore the association between the SIRT1 expression and the clinical characteristics, the responsiveness to chemotherapy and prognosis in Non-small cell lung cancer (NSCLC).

**Methods:**

We enrolled 295 patients with inoperable advanced stage of NSCLC, namely, stage III (A+B) and IV NSCLC. All patients had received platinum-based chemotherapy after diagnosis and the chemotherapy response were evaluated. All patients were followed up for overall survival (OS) and progression free survival (PFS). *In* *vitro*, H292 cells were tranfected with SIRT1 small interfering RNA (siRNA). The cell biological behaviors and chemosensitivity to cisplatin treatment were studied. The *in* *vivo* tumorgenesis and metastasis assays were performed in nude mice.

**Results:**

We found that the SIRT1 expressions were significantly associated with the tumor stage, tumor size and differentiation status. Patients with high SIRT 1 expressions had a significantly higher chance to be resistant to chemotherapy than those with low SIRT 1 expression. Patients with high expression of SIRT1 had significantly shorter OS and DFS than those with low expression. Cox analyses confirmed that the SIRT 1 expression was a strong predictor for a poor OS and PFS in NSCLC patients underwent Platinum-based chemotherapy. *In* *vitro* studies revealed that the reduced expression SIRT 1 by siRNA technique significantly inhibited cell proliferation, migration and invasion. More importantly, SIRT1 si-RNA significantly enhanced the chemosensitivity of H292 cells to cisplatin treatment. The *in* *vivo* tumorgenesis and metastasis assays showed that SIRT1 knockdown dramatically reduced the tumor volume and the metastatic ability in nude mice.

**Conclusion:**

Collectively, our data suggest that the SIRT1 expression may be a molecular marker associated with the NSLCLC clinical features, treatment responsiveness and prognosis of advanced NSCLC.

## Introduction

Lung cancer is one of the deadliest cancers worldwide, with the highest incidence and mortality amongst all cancers [1]. Non-small cell lung cancer (NSCLC) accounts for approximately 80% of primary lung cancers [2]. The prognosis of NSCLC is very poor and the 5-year survival rate of lung cancer is below 20% [2]. Platinum-based chemotherapy is the standard first-line chemotherapy for advanced NSCLC, however, drug resistance is remains a major factor influencing the clinical outcome of patients [3–5]. Significant variability in prognosis has still been observed in patients with similar clinical features [3,6,7]. The identification of new maker predicting the chemotherapy response is important for to improve the prognosis of patients with NSCLC. 

Sirtuin 1 (SIRT 1) is a member of Sirtuin family, the mammalian homologues of the silent information regulator 2 first discovered in *Saccharomyces cerevisiae* as an NAD^+^-dependent histone deacetylase [8]. SIRT1 has been shown to control cell cycle, proliferation and senescence [9–12]. Up-regulation of SIRT1 has been reported in various human malignancies including prostate cancer, breast cancer, lymphoma, colon cancer, and gastric cancer [11,13–16]. In contrast, SIRT1 inhibitor can induces senescence-like growth arrest with attenuated Ras-MAPK signaling in human cancer cells [17], suggesting that SIRT1 inhibitors may have anticancer potential. Down-regulation of SIRT1 induces apoptosis and enhances radiation sensitization in A549 lung cancer cells [18]. Activation of SIRT1 significantly promotes tumor cell migration and metastasis of breast cancer in mice [15]. Moreover, a recent study in hepatocellular carcinoma (HCC) showed that the over-expression of SIRT1 promoted tumorigenesis and resistance to chemotherapeutical agent and sorafenib [19].however, the association between SIRT1 expression and the clinical characteristics, especially the responsiveness to chemotherapy and prognosis in NSCLC remain largely unknown. 

## Methods

### Patient enrollment

A total of 295 patients with inoperable advanced stage of NSCLC, namely, stage III (A+B) and IV NSCLC confirmed cytologically or histologically were enrolled into this study. The staging system we used was the 7th edition of the TNM system [20]. All patients had received platinum-based chemotherapy after diagnosis (Table 1). The inclusion and exclusion criteria were described previously elsewhere [21]. The study was approved by the ethics committees of our hospital and written informed consent was obtained from each participant. 

**Table 1:** Patient characteristics between chemotherapy responder and non-responders.

*Characteristics Patient*	*Responder (n=110)*	*Non-responder (n=185)*	*P*
***Age(years***)	*57.4±6.6*	*56.4±7.2*	*NS*
***Gender***			
*Male*	*58*	*96*	*NS*
*Female*	*52*	*89*	
***Smoke status***			
*Non-smokers*	*54*	*56*	*<0.001*
*Smoker*	*56*	*129*	
***Histology***			
*Squamous cell carcinoma*	*54*	*93*	*NS*
*Adenocarcinoma*	*56*	*92*	
***Stage***			
*IIIA*	*47*	*38*	*<0.001*
*IIIB*	*50*	*82*	
*IV*	*13*	*65*	
***Differentiation***			
*Well*	*46*	*35*	*<0.001*
*Moderate*	*43*	*101*	
*Poor*	*21*	*49*	
***Chemotherapyregimens***			
*DDP/CBP+TAX/TXT/DOC*	*45*	*56*	*NS*
DDP/CBP+*GEM*	*33*	*69*	
DDP/CBP+*NVB*	*32*	*60*	

NS not significance, DDP cisplatin, CBP carboplatin, TAX taxol/paclitaxel, TXT taxetere, DOC docetaxel, GEM gemcitabine, NVB vinorelbine

### Chemotherapy regimens and therapeutic effect evaluation

Patient responses to treatment were determined after four cycles by the WHO criteria [21], which classify the response into four categories: complete response (CR), partial response (PR), stable disease (SD), and progressive disease (PD). CR was defined as complete disappearance of all measurable lesions. PR required at least 50% reduction in measurable lesions. Patients with SD had less than a 50% decrease or no more than a 25% increase in the size of measurable lesions. PD was assigned to patients when measurable lesions increased by more than 25% or new lesions appeared. 

### Outcome Data

Overall survival (OS) and progression free survival (PFS) were the end points in this study. OS was calculated from the date of chemotherapy to the date of last follow-up or death from any cause. PFS was defined as the interval between the date of chemotherapy and the date of confirmed relapse. 

### Immunohistochemical analysis and evaluation

Tumor samples were obtained from the biopsy before the start of chemotherapy. The tissues were fixed, paraffin embedded, and cut to 5-μm-thick sections for immunohistochemistry. Briefly, the slides were incubated with SIRT 1 primary antibody (1:200, Santa Cruz Biotechnology, Santa Cruz, CA). The immunoreactive products were visualized by the catalysis of 3,3′-diaminobenzidine (DAB). SIRT1 expression were classified semiquantitatively by immunoreactive score (IRS) according to the method described previously [22]. SIRT 1 staining was defined as low expression (IRS: 0–5) and high expression (IRS: 6–12).

### Tumor Cell line Culture and small interfering RNA (siRNA) transfection

A human NSCLC cell line , H292, was cultured to 75% confluence before small interfering RNA (siRNA) transfection. siRNA-annealed oligonucleotide duplexes for SIRT1 (Sequence 5'->3' sense: GCAAUAGGCCUCUUAAUUAtt; antisense: UAAUUAAGGCCUAUUGCtt) and control si-RNA purchased from GenePharma (Shanghai, China) were transfected by siLentFect Lipid Reagent (Bio-Rad, Hercules, CA, USA) for 36 hours according to the manufacturer’s instructions. 

### Western Blot Analysis

After transfection, the expression of SIRT1 expression in treated was detected by cells by western blot assay. After immunoblot analysis, membranes were immunoblotted with SIRT 1 antibody (1:1000, Santa Cruz Biotechnology, Santa Cruz, CA). Membranes were then washed and incubated with a secondary antibody coupled to horseradish peroxidase.

### MTT Cell Proliferation Assay

Cells were seeded in a 96-well plate right after si-RNA transfection. The medium were discarded and 100 μl of dimethyl sulfoxide was added to each well. Absorbance was measured at a wavelength of 490 nm (with 630 nm as the reference wavelength) using an ELISA microplate reader (Bio-Rad, Hercules, CA, USA). Assays were repeated at least three times. 

### Cell invasion and migration assay

The cell invasion and migration ability was evaluated using transwell inserts with 8 µm pores (BD Biosciences, San Jose, CA, USA). For invasion assay, 2×10^5^ cells in serum free medium were added to each upper compartment of the chamber pre-coated with matrigel matrix (BD Biosciences, San Jose, CA, USA). After incubation for 48 hours, noninvasive cells were removed from the upper surface of the transwell membrane, and migrated cells were fixed with methanol, stained with Giemsa and photographed under the microscope. For migration assay, 2×10^5^ cells were placed into the top chamber without matrigel matrix pre-coated. Finally, the cells in lower compartment of the chamber that had invaded to the basal side of the membrane were counted using a light microscope in 5 random visual fields (×200) [23].

### Cisplatin cytotoxicity assay

H292 cells were seeded at 1×10^4^ cells per well in 96-well plates, and were transfected by SIRT 3 siRNA and control siRNA for 48h, and subsequently exposed to cisplatin at final concentrations of 0.5, 1.0, 2.0, 4.0 or 8.0 ug/ml for 24h in triplicate wells. Cell survival was determined using a previously described colorimetric MTT assay. Mean cell viability was calculated by the ratio of absorbance units of transfected cell samples to the mean absorbance units of the control cell samples. All the experiments were repeated at least three times. The IC50 value is defined as concentration of cisplatin that is required for a 50% reduction in absorbance calculated from the growth curves.

### *In vivo* tumorgenesis and metastasis assay

For *in vivo* tumorgenesis assay, a total of 10 nude mice were inoculated subcutaneously with H292 cells (1×10^6^/animal) transfected with SIRT1 siRNA (n=5) and control siRNA (n=5). Tumor size was assessed by caliper measurements twice a week. Mice were sacrificed four weeks after tumor cell inoculation and tumors were excised for further analysis. Tumor volumes were calculated using the following formula: (cubic millimeters) = (length × width2) × 0.5. Mouse weights were recorded every 2 days [24] .The tumor invasion in liver, lung, bone, prestate and brain were detected by histological staining. For in vivo metastasis assay, H292 cells (1×10^6^/animal) treated with transfected with SIRT1 siRNA and control siRNA were injected into nude mice (n=5 in each group). All mice were sacrificed at 14 days to obtain lung lobes. Lung tissue were sliced and underwent the hematoxylin and eosin (HE) stain. The tumor site in the lung slice were observed under microscopy and counted in each field. 

### Statistical Analyses

Association between SIRT1 expression status and clinical parameters were studied using chi-square test, or independent t tests. Multivariate analysis was performed using the Cox proportional hazards model selected in forward stepwise. The odds ratios (OR) and 95% confidence intervals (CIs) were calculated. Survival was analyzed using the Kaplan–Meier method. The log-rank test was used to analyze survival differences. The data in proliferation rate, migration, invasiveness, cisplatin cytotoxicity assays and the *in vivo* tumorgenesis and metastasis assays were performed in nude mice between SIRT1 si-RNA and control si-RNA treated cells were compared by using *t* tests. P < 0.05 was considered statistically significant. All analyses were performed by using SAS software (version 9.2, USA). 

## Results

### NSCLC Patient Characteristics

Among all enrolled patients, 110 patients were assigned as chemotherapy responder (CR + PR) and 185 were non-responder (SD + PD). No significant differences were noted in mean age, gender distribution, histology type and chemotherapy agent between the chemotherapy responder and non-responders (table 1). However, those non-responders had higher a smoker number, higher tumor stage, poorer differentiation than responders (all P<0.001). 

### The association between SIRT1 expression and clinical characteristics of NSCLC

**Figure 1:**
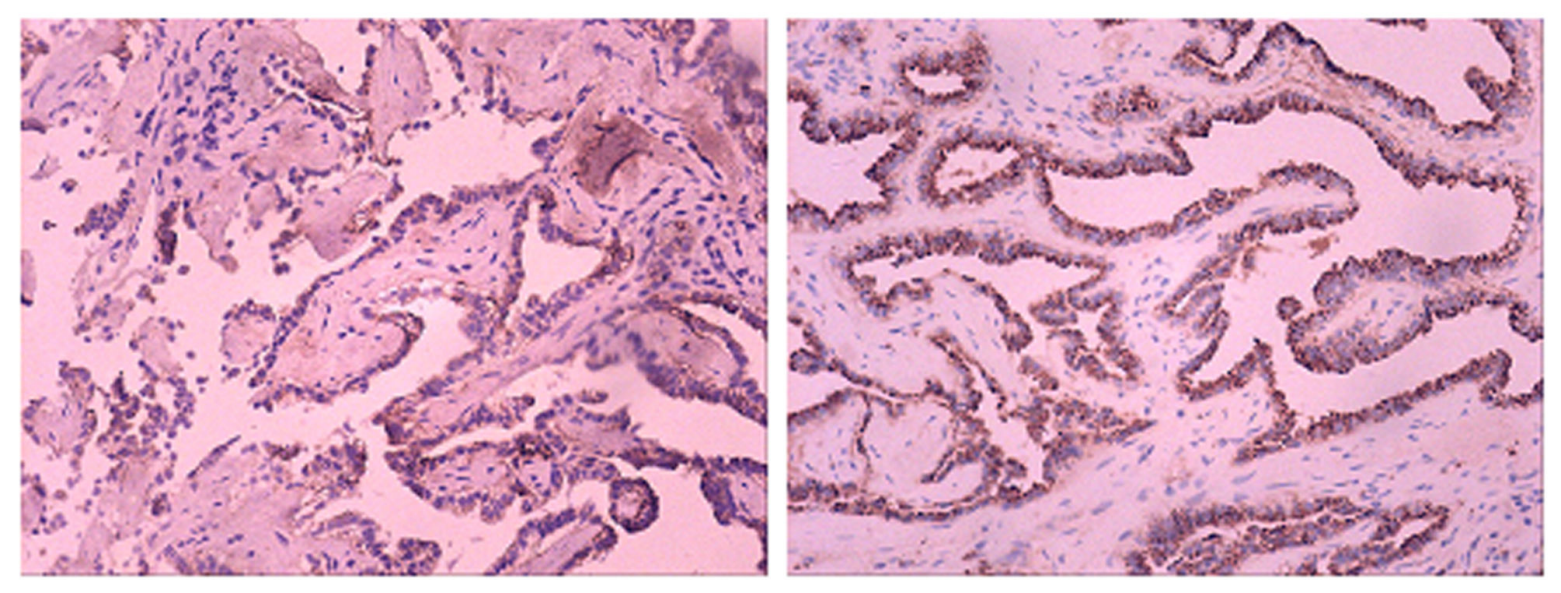
Representative figures of SIRT1 staining in 2 patients with adenocarcinoma NSCLC. Left: low SIRT 1 expression; right: high SIRT 1 expression.

Representative figures of SIRT1 staining are shown in Figure 1. SIRT1 was mainly expressed in the cytoplasm of cancer tissues. Table 2 shows the association between SIRT1 expression status and clinical characteristics of NSCLC patients. We found that SIRT1 expression were significantly associated with the tumor stage, tumor size and differentiation status (table 2), but not related to age, smoke status, histology and chemotherapy regimens (data not shown). 

**Table 2:** The association between SIRT1 and clinical characteristics ob NSCLC.

*Clinical features*	*High SIRT 1evel*		*Low SIRT1 level*		*OR*	*95%CI*		*P*
***Tumor Stage***								
*IIIA*	*36*	*21.69%*	*49*	*37.98%*	*1.00*			
*IIIB*	*77*	*46.39%*	*55*	*42.64%*	*1.91*	*1.10*	*3.31*	*0.02*
*IV*	*53*	*31.93%*	*25*	*19.38%*	*2.89*	*1.52*	*5.48*	*<0.001*
***Tumor differentiation***								
*Well*	*47*	*28.31%*	*65*	*50.39%*	*1.00*			
*Moderate*	*79*	*47.59%*	*51*	*39.53%*	*2.14*	*1.28*	*3.58*	*<0.001*
*Poor*	*40*	*24.10%*	*13*	*10.08%*	*4.26*	*2.05*	*8.83*	*<0.001*

### SIRT1 expression affected the chemotherapy response in advanced NSCLC

The SIRT1 high expressions were observed in 137 of 185 non-responders (74.1%) but only in 29 of 110 responders (26.4%). Table 3 shows the association between the SIRT 1 expression and chemotherapy response status. Patients with high SIRT1 expression had a significantly higher chance to be resistant to chemotherapy than those with low SIRT 1 expression (OR=7.97, 95%CI: 4.66-10.63, P<0.001, table 3). 

**Table 3:** Shows the association between the SIRT 1 expression and chemotherapy response status.

*SIRT1 expression*	*Non-responder*	*%*	*Responder*	*%*	*OR*	*95%CI*		*P*
*Low SIRT1*	*48*	*25.95%*	*81*	*73.64%*	*1.00*			
*High SIRT1*	*137*	*74.05%*	*29*	*26.36%*	*7.97*	*4.66*	*10.63*	*<0.001*

### The SIRT1 expression predicted the prognosis of NSCLC

**Figure 2:**
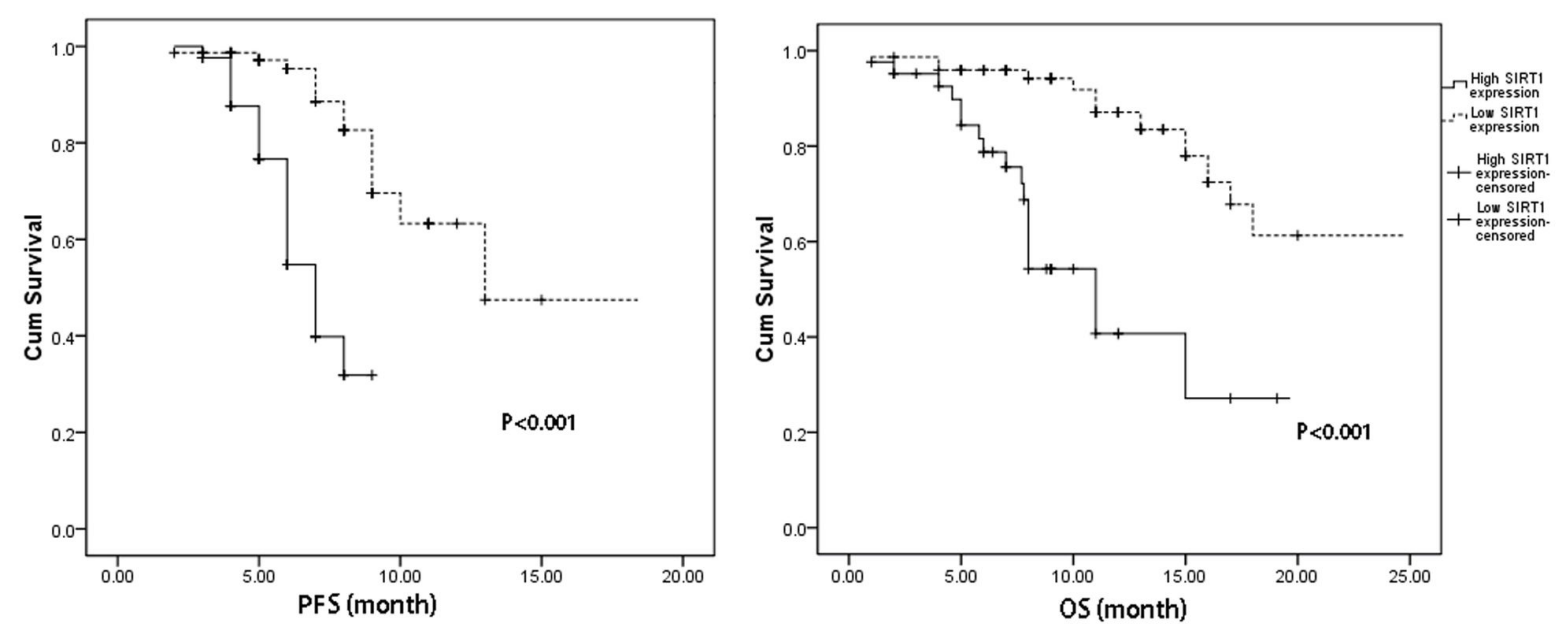
Kaplan–Meier survival curves analyses by SIRT1 expression. Figure 2a: the PFS analyses by SIRT1 expression status (high SIRT1 expression vs. low SIRT1 expression, P<0.001) ; 2b: the OS analyses by SIRT1 expression status ((high SIRT1 expression vs. low SIRT1 expression, P<0.001).

The associations between the clinical variables and PFS as well as OS were studied by log-rank test. Patients with high expression of SIRT1 expressions had markedly shorter PFS and OS than those with low SIRT1 expressions (6.8±2.1 vs. 8.8±2.4, months, P<0.001 and 11.1±7.2 vs.17.1±2.8, months, P <0.001, respectively). Kaplan–Meier survival curves were shown in Figure 2a and 2b. 

Univariate and multivariate analyses were performed by using Cox proportional hazard model to evaluate the impact of SIRT1 expression and other pathological factors on the prognosis of NSCLC (Table 4). Univariate analysis showed four statistically significant variables associated with the prognosis of NSCLC: tumor stage (P<0.001), smoker (P=0.024), tumor differentiation (P=0.003) and SIRT1 expression (P<0.001). The multivariate analyses confirmed that tumor stage, tumor differentiation and SIRT 1 expression predicted poor PFS and OS in NSCLC patients (table 4). SIRT1 had significantly higher chance of having poor PFS (HR=3.32, P<0.001) and OS (HR=2.48, P<0.001). 

**Table 4:** Multivariate Cox proportional regression analysis on PFS and OS of NSCLC patients.

	*HR(PFS)*	*95%CI(PFS)*	*P(PFS)*	*HR(OS)*	*95%CI(OS)*	*P(OS)*
***SIRT1 expression status***						
*Low expression*	*1*		*<0.001*	*1*		*0.003*
*High expression*	*3.32*	*2.32–4.83*		*2.48*	*2.28–3.76*	
***Tumor stage***						
*IIIA*	*1*		*0.0021*	*1*		*0.009*
*IIIB+IV*	*2.63*	*2.65–4.47*		*1.81*	*1.98–3.36*	
**tumor differentiation**						
*Well*	*1*		*<0.001*	*1*		*<0.001*
*Moderate +poor*	*3.13*	*2.03–4. 82*		*2.021*	*1.64–311*	

### SIRT 1 regulated the proliferation, migration and invasion of H292 cells

**Figure 3:**
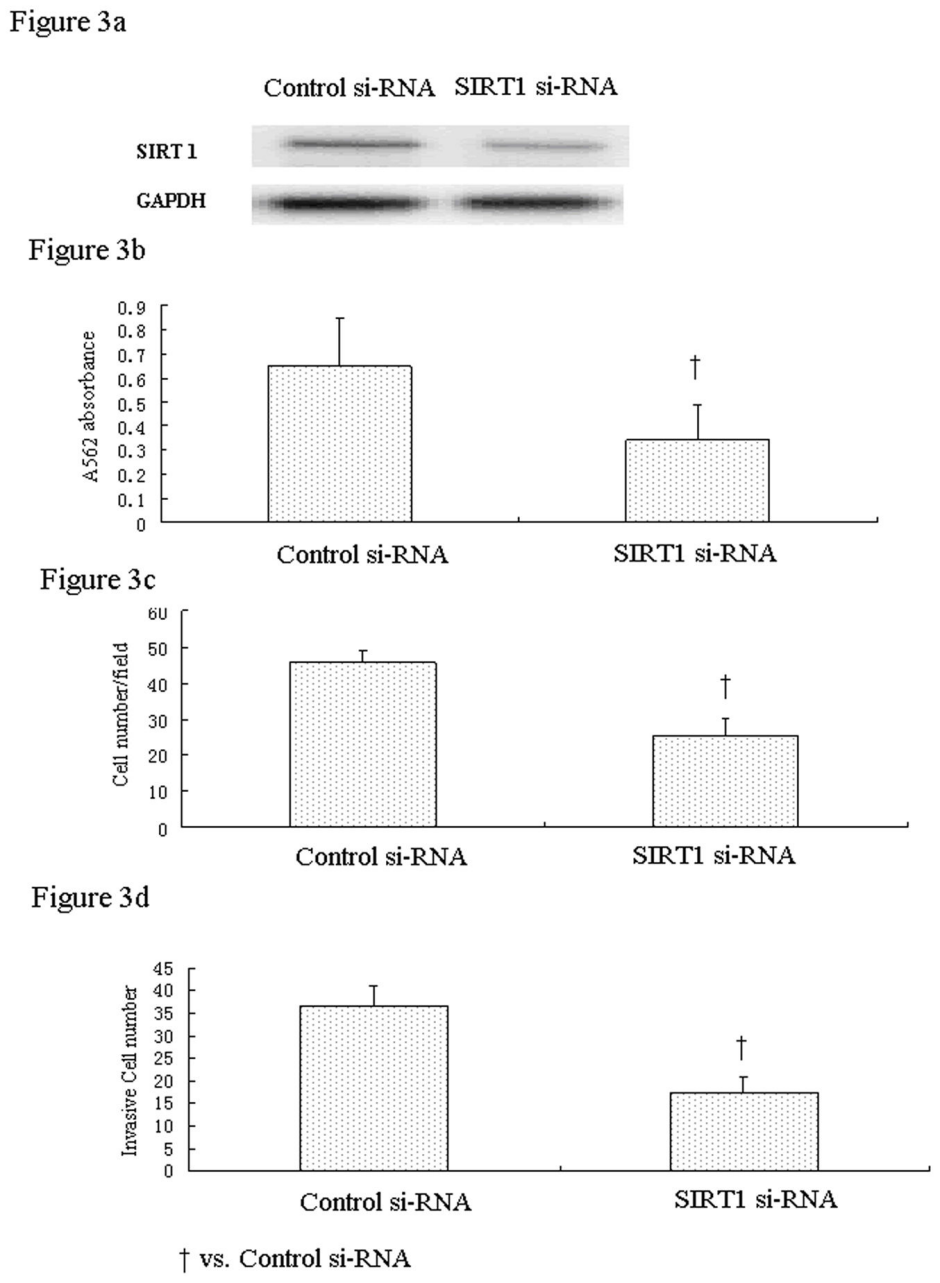
The proliferation, migration and invasiveness of NSCLC cells infected with SIRT 1 siRNA and control siRNA. a. the SRIT1 expression after SIRT1 si-RNA transfection in H292 cells. b the absorbance at 562nm between cells infected with SIRT 1 si-RNA and control si-RNA (P<0.001). c the cell numbers of migrated cells infected with SIRT 1 si-RNA and control si-RNA (P<0.001). d the cell numbers of invasive cells infected with SIRT 1 si-RNA and control si-RNA (P<0.001).

Western blot results showed that the SIRT 1 protein expressions in H292 cells were significantly inhibited by SIRT1 si-RNA silencing technique (Figure 3a). The cell proliferation assays revealed the cell growth rate was significantly inhibited in H292 cells after SIRT1 si-RNA transfection by 47.3% compared to cells treated with control si-RNA (Figure 3ba). Cell migration assay showed that SIRT1 knockdown significantly decreased the migrated cell numbers by 43.8% (Figure 3c). Furthermore, silencing of SIRT1 gene dramatically inhibited the invasive ability of H292 cells by 57.6% (Figure 3d). 

### The SIRT1 si-RNA transfection induced chemosensitivity to cisplatin in cultured cancer cells

**Figure 4:**
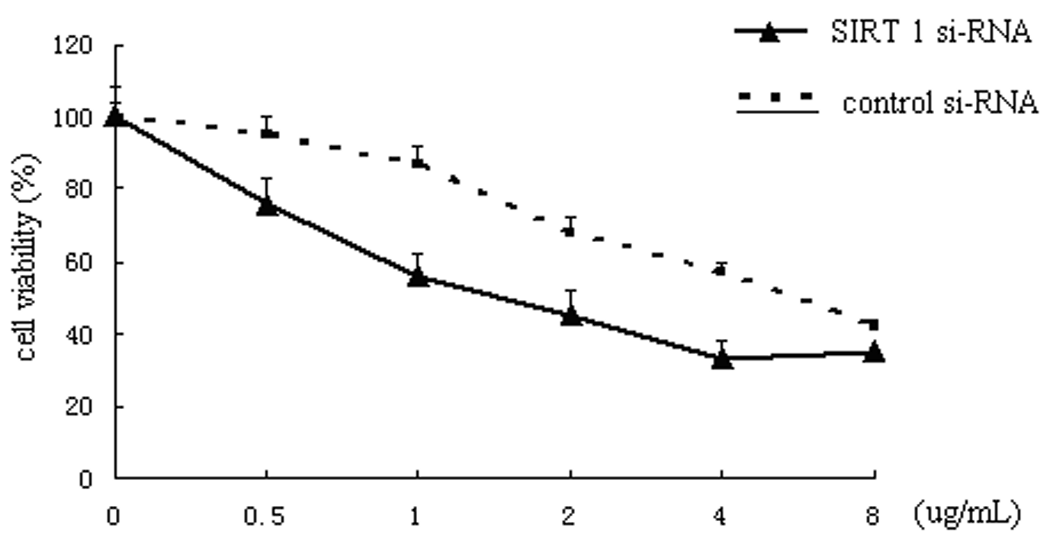
SIRT 1 siRNA-induced enhancement of chemosensitivity to cisplatin. Sensitivity curves of the H292 cell line toward cisplatin via MTT assay. Growth inhibition of SIRT1 siRNA-transfected cells was observed after exposure to different concentrations of cisplatin (0-8ug/mL).

Exposure of SIRT1 si-RNA-transfected H292 cells to different concentrations of cisplatin induced significant proliferative inhibition. When the concentration of cisplatin was 8 ug/ml; the viability of SIRT1 siRNA-transfected cells showed no significant difference compared with those of the control siRNA-transfected and untreated cells (Figure 4). Meanwhile, the IC50 concentration of cisplatin for cancer cells decreased significantly from 6.34 ug/ml in control cells and 1.61 ug/ml in SIRT1 si-RNA-transfected cells, indicating that SIRT1 silencing significantly enhanced the chemosensitivity of H292 cells to cisplatin treatment. 

### The *in vivo* tumorgenesis and metastasis assay

**Figure 5:**
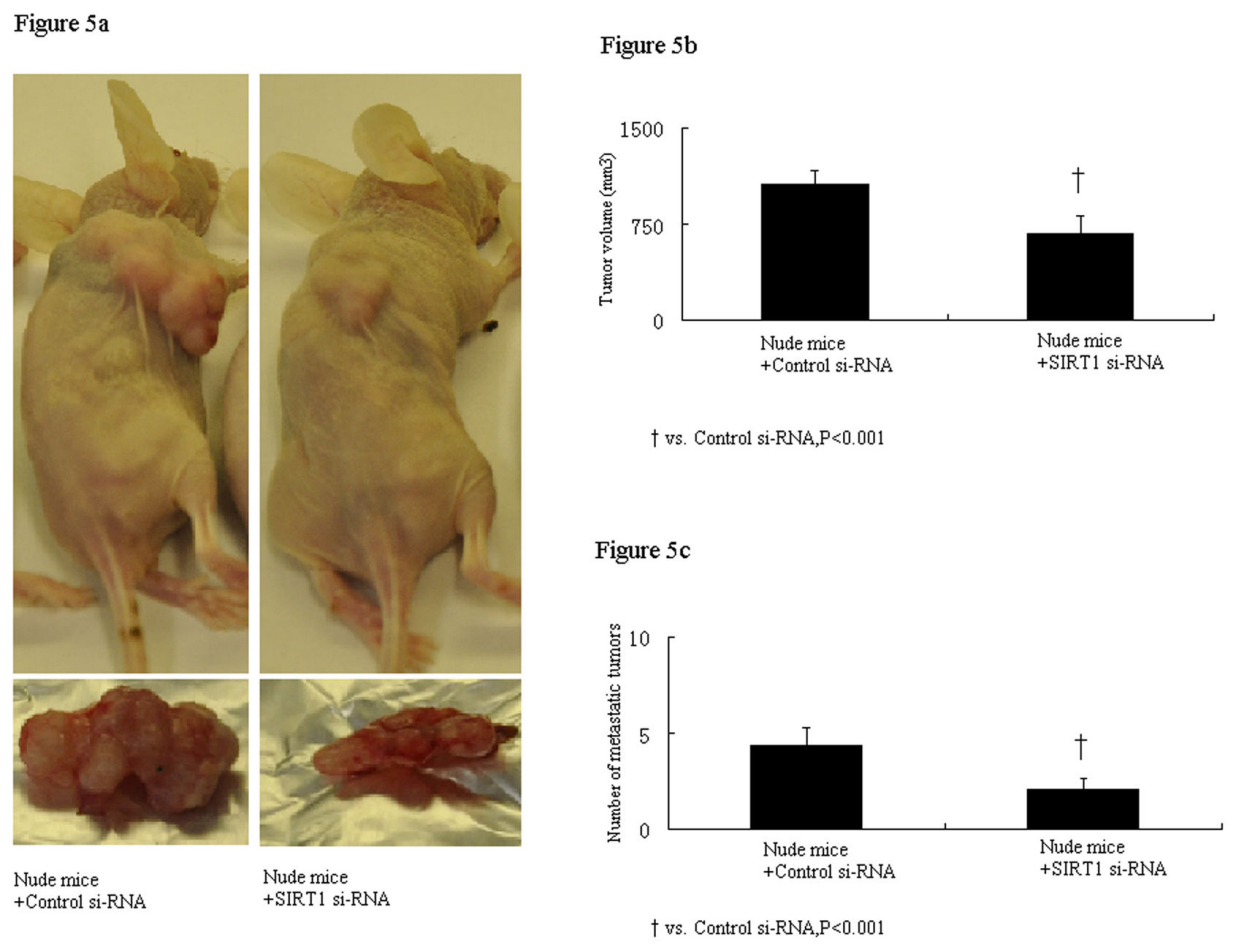
The in vivo tumorgenesis assay and metastasis assay in nude mice. Nude mice inoculated with SIRT1 si-RNA-transfected cells had markedly reduced tumor volume compared to mice received cells transfected with control si-RNA transfection (730±141vs. 1153±112, mm3 , P<0.001, Figure 5a and 5b).The in vivo metastasis assay shows the inoculation with SIRT1 si-RNA-transfected cells in nude mice had dramatically reduced numbers of metastatic tumors in lung tissues than control si-RNA-transfected cells (4.4±0.9 vs. 2.1±0.5, P<0.001, Figure 5c).

The *in vivo* tumorgenesis assay showed that nude mice inoculated subcutaneously with SIRT1 si-RNA-transfected cells had markedly reduced tumor volume compared to mice received cells transfected with control si-RNA transfection (730±141vs. 1153±112, mm^3^ , P<0.001, Figure 5a and 5b).The *in vivo* metastasis assay shows the inoculation with SIRT1 si-RNA-transfected cells in nude mice had dramatically reduced numbers of metastatic tumors in lung tissues than control si-RNA-transfected cells (4.4±0.9 vs. 2.1±0.5, P<0.001, Figure 5c).

## Discussion

In the present study, we investigated the role of SIRT 1 expression in the clinical features, responsiveness to platinum-based chemotherapy and prognosis of NSCLC patients receiving Platinum-based chemotherapy. We found that SIRT1 expression were significantly associated with the tumor stage, size and differentiation status. Patients with high SIRT1 expression had a significantly higher chance to be resistant to chemotherapy than those with low SIRT 1 expression. SIRT1 expression status affected the PFS as well as OS as well. Cox analyses confirmed that SIRT 1 expression was a strong predictor for a poor OS and PFS in NSCLC patients underwent platinum-based chemotherapy. Also we studied the effect of SIRT1 on the biological behaviors of NSCLC cells. *In vitro* studies revealed that inhibition of SIRT 1 by siRNA technique significantly inhibited cell proliferation, migration and invasion. More importantly, we found that the SIRT1 si-RNA significantly enhanced the chemosensitivity of H292 cells to cisplatin treatment. The *in vivo* studies in nude mice confirmed SIRT1 is involved in the regulation of tumorgenesis and metastases. Collectively, our data suggest that the SIRT1 may be a molecular marker associated with the NSLCLC clinical features, treatment responsiveness and prognosis of advanced NSCLC. 

SIRT1 is the best characterized member within the family of sirtuins with regard to life span and age-related disease. SIRT1 is expressed in all mammalian cells and was originally identified as a nuclear protein. However, recent studies showed that subcellular localization of SIRT1 differs from cell to cell. While some cells showed nuclear localization of SIRT1, others expressed it either both in the nucleus and in the cytoplasm or in the cytoplasm alone [25]. In our study, we found that the SIRT 1 expression were predominantly in the cytoplasm in NSCLC cancer tissues. 

SIRT1 is considered a novel anti-aging protein involved in regulation of cell cycle arrest, cellular senescence, proliferation, and resistance to oxidative stress and apoptosis [26] [27] [28] [29] [30]. However, the role of SIRT1 in tumorogenesis is very controversial. Increased expression of SIRT1 has been reported in numerous types of cancer in human such as prostate cancer [14], leukemia [31], primary colon cancer [32], and breast cancer [33]. However, other publications have indicated that SIRT1 has tumor-suppressive functions *in vivo*. For instance, activation of SIRT1 by resveratrol was shown to limit cell growth and reduce tumour formation in BRCA1-deficient tumour cells as well asTrp53+/−;Sirt1+/− mice [34]. SirT1 has also been shown to inhibit androgen receptor-dependent cell proliferation in prostate tumor cells [35]. In the present study, we found that the SIRT1 expression is associated with higher tumor stage and poorer differentiation status, and poor prognosis, indicating that at least in advanced NSCLC, SIRT1 expression was parallel with the disease severity. Thus, the effect of SIRT1 may vary according to the cell type, stage of tumor development, and accompanying mutation status of tumor related genes.

Currently, there is no study with regard to the effect of SIRT1 expression level on the chemotherapy response in NSCLC tumors. Our study was the first to investigate the association between SIRT1 expression and the responsiveness to chemotherapy in a clinical setting. We found that high SIRT 1 expression had a significantly higher chance to be resistant to chemotherapy than those with low SIRT 1 expression. In vitro, reduced expression SIRT 1 by siRNA technique significantly enhanced the chemosensitivity of H292 cells to cisplatin treatment. This is consistent with a previous study, which reported that overexpression of SIRT1promoted tumorigenesis and resistance to chemotherapeutical agent and sorafenib in liver cancer [19]. A previous study also showed that SIRT1 confers hypoxia-induced radioresistance via the modulation of c-Myc stabilization on hematoma cells [36]. Knockdown of SirT1 expression enhanced radiosensitivity and radiation-induced apoptosis in glioma CD133-positive cells [37]. SIRT1 knockout cells were more susceptible to cell death induction following ionizing radiation and cisplatin [38]. Collectively, these studies suggest that SIRT1 could serve as a novel potent pharmacological target of cancer chemotherapy.

Our *in vitro* data also showed that SIRT1 siRNA intervention significantly inhibited the Proliferation, Migration and Invasion of cultured NSCLC Cells. This observation was consistent with a previous study, which reported that SIRT1 expression improves cell survival, which could be a sign that they may in fact promote tumorigenesis [39,40].  Another study in liver cancer cell lines revealed that overexpression of SIRT1 promoted mitotic entry of liver cells, cell growth and proliferation and inhibited apoptosis. SIRT1 enhances matrix metalloproteinase-2 expression and tumor cell invasion in prostate cancer cells [41].

Several limitations should be addressed in this study. Firstly, the sample size of clinical part in this study was relatively small and enrolled only Chinese patients. To better elucidate the role that SIRT1 played in NSCLC, further study with larger sample size and multiple ethnicity is warranted. Secondly, we did not yet explore the molecular mechanism under which SIRT1 regulated the cell biological behaviors and its influence on chemosensitivity to anti-tumor medications.

## References

[1] Subramaniam S, Thakur RK, Yadav VK, Nanda R, Chowdhury S et al. (2013) Lung cancer biomarkers: State of the art. J Carcinog 12: 3. doi:10.4103/1477-3163.107958. PubMed: 23599685. 23599685 PMC3622361

[2] Tsim S, O'Dowd CA, Milroy R, Davidson S (2010) Staging of non-small cell lung cancer (NSCLC): a review. Respir Med 104: 1767-1774. doi:10.1016/j.rmed.2010.08.005. PubMed: 20833010. 20833010

[3] Kosmidis P (2002) Chemotherapy in NSCLC: historical review. Lung Cancer 38 Suppl 3: S19-S22. doi:10.1016/S0169-5002(02)00261-1. PubMed: 12468139. 12468139

[4] Hildebrandt MA, Gu J, Wu X (2009) Pharmacogenomics of platinum-based chemotherapy in NSCLC. Expert Opin Drug Metab Toxicol 5: 745-755. doi:10.1517/17425250902973711. PubMed: 19442035. 19442035 PMC3417337

[5] Gridelli C, Ferrara C, Del Gaizo F, Guerriero C, Nicolella D et al. (2002) Chemotherapy of advanced NSCLC in the elderly. Tumori 88: S143-S144. PubMed: 11989910. 11989910 10.1177/030089160208800142

[6] Stuschke M, Pöttgen C (2010) Chemotherapy: Effectiveness of adjuvant chemotherapy for resected NSCLC. Nat Rev Clin Oncol 7: 613-614. doi:10.1038/nrclinonc.2010.165. PubMed: 20981122. 20981122

[7] Almeida GM, Duarte TL, Farmer PB, Steward WP, Jones GD (2008) Multiple end-point analysis reveals cisplatin damage tolerance to be a chemoresistance mechanism in a NSCLC model: implications for predictive testing. Int J Cancer 122: 1810-1819. PubMed: 18074354. 18074354 10.1002/ijc.23188

[8] Sauve AA, Wolberger C, Schramm VL, Boeke JD (2006) The biochemistry of sirtuins. Annu Rev Biochem 75: 435-465. doi:10.1146/annurev.biochem.74.082803.133500. PubMed: 16756498. 16756498

[9] Fusco S, Maulucci G, Pani G (2012) Sirt1: def-eating senescence? Cell Cycle 11: 4135-4146. doi:10.4161/cc.22074. PubMed: 22983125. 22983125 PMC3524209

[10] Jang KY, Noh SJ, Lehwald N, Tao GZ, Bellovin DI et al. (2012) SIRT1 and c-Myc promote liver tumor cell survival and predict poor survival of human hepatocellular carcinomas. PLOS ONE 7: e45119. doi:10.1371/journal.pone.0045119. PubMed: 23024800. 23024800 PMC3443243

[11] Chen W, Bhatia R (2013) Roles of SIRT1 in leukemogenesis. Curr Opin Hematol, 20: 308–13. PubMed: 23519155. 23519155 10.1097/MOH.0b013e328360ab64 PMC3808881

[12] Li X (2013) SIRT1 and energy metabolism. Acta Biochim Biophys Sin (Shanghai) 45: 51-60. doi:10.1093/abbs/gms108. 23257294 PMC3527007

[13] Lee H, Kim KR, Noh SJ, Park HS, Kwon KS et al. (2011) Expression of DBC1 and SIRT1 is associated with poor prognosis for breast carcinoma. Hum Pathol 42: 204-213. doi:10.1016/j.humpath.2010.05.023. PubMed: 21056897. 21056897

[14] Huffman DM, Grizzle WE, Bamman MM, Kim JS, Eltoum IA et al. (2007) SIRT1 is significantly elevated in mouse and human prostate cancer. Cancer Res 67: 6612-6618. doi:10.1158/0008-5472.CAN-07-0085. PubMed: 17638871. 17638871

[15] Suzuki K, Hayashi R, Ichikawa T, Imanishi S, Yamada T et al. (2012) SRT1720, a SIRT1 activator, promotes tumor cell migration, and lung metastasis of breast cancer in mice. Oncol Rep 27: 1726-1732. PubMed: 22470132. 22470132 10.3892/or.2012.1750

[16] Bae HJ, Chang YG, Noh JH, Kim JK, Eun JW et al. (2012) DBC1 does not function as a negative regulator of SIRT1 in liver cancer. Oncol Lett 4: 873-877. PubMed: 23162614. 23162614 10.3892/ol.2012.875 PMC3499483

[17] Ota H, Tokunaga E, Chang K, Hikasa M, Iijima K et al. (2006) Sirt1 inhibitor, Sirtinol, induces senescence-like growth arrest with attenuated Ras-MAPK signaling in human cancer cells. Oncogene 25: 176-185. PubMed: 16170353. 16170353 10.1038/sj.onc.1209049

[18] Sun Y, Sun D, Li F, Tian L, Li C et al. (2007) Downregulation of Sirt1 by antisense oligonucleotides induces apoptosis and enhances radiation sensitization in A549 lung cancer cells. Lung Cancer 58: 21-29. doi:10.1016/j.lungcan.2007.05.013. PubMed: 17624472. 17624472

[19] Chen HC, Jeng YM, Yuan RH, Hsu HC, Chen YL (2012) SIRT1 promotes tumorigenesis and resistance to chemotherapy in hepatocellular carcinoma and its expression predicts poor prognosis. Ann Surg Oncol 19: 2011-2019. doi:10.1245/s10434-011-2159-4. PubMed: 22146883. 22146883

[20] Goldstraw P, Crowley J, Chansky K, Giroux DJ, Groome PA et al. (2007) The IASLC Lung Cancer Staging Project: proposals for the revision of the TNM stage groupings in the forthcoming (seventh) edition of the TNM Classification of malignant tumours. J Thorac Oncol 2: 706-714. doi:10.1097/JTO.0b013e31812f3c1a. PubMed: 17762336. 17762336

[21] Wu F, Hu N, Li Y, Bian B, Xu G et al. (2012) Galectin-3 genetic variants are associated with platinum-based chemotherapy response and prognosis in patients with NSCLC. Cell Oncol (Dordr) 35: 175-180. 10.1007/s13402-012-0075-7 PMC12994991 22476961

[22] Remmele W, Stegner HE (1987) [Recommendation for uniform definition of an immunoreactive score (IRS) for immunohistochemical estrogen receptor detection (ER-ICA) in breast cancer tissue]. Pathologe 8: 138-140. PubMed: 3303008. 3303008

[23] Zhou C, Liu G, Wang L, Lu Y, Yuan L et al. (2013) MiR-339-5p Regulates the Growth, Colony Formation and Metastasis of Colorectal Cancer Cells by Targeting PRL-1. PLOS ONE 8: e63142. doi:10.1371/journal.pone.0063142. PubMed: 23696794. 23696794 PMC3656035

[24] Dai C, Zhang B, Liu X, Ma S, Yang Y et al. (2013) Inhibition of PI3K/AKT/mTOR pathway enhances temozolomide-induced cytotoxicity in pituitary adenoma cell lines in vitro and xenografted pituitary adenoma in female nude mice. Endocrinology 154: 1247-1259. doi:10.1210/en.2012-1908. PubMed: 23384836. 23384836

[25] Sundaresan NR, Pillai VB, Gupta MP (2011) Emerging roles of SIRT1 deacetylase in regulating cardiomyocyte survival and hypertrophy. J Mol Cell Cardiol 51: 614-618. doi:10.1016/j.yjmcc.2011.01.008. PubMed: 21276800. 21276800 PMC3442925

[26] Kong S, McBurney MW, Fang D (2012) Sirtuin 1 in immune regulation and autoimmunity. Immunol Cell Biol 90: 6-13. doi:10.1038/icb.2011.102. PubMed: 22105513. 22105513

[27] Metoyer CF, Pruitt K (2008) The role of sirtuin proteins in obesity. Pathophysiology 15: 103-108. doi:10.1016/j.pathophys.2008.04.002. PubMed: 18599274. 18599274

[28] Mantel C, Broxmeyer HE (2008) Sirtuin 1, stem cells, aging, and stem cell aging. Curr Opin Hematol 15: 326-331. doi:10.1097/MOH.0b013e3283043819. PubMed: 18536570. 18536570 PMC2653857

[29] Zschoernig B, Mahlknecht U (2008) SIRTUIN 1: regulating the regulator. Biochem Biophys Res Commun 376: 251-255. doi:10.1016/j.bbrc.2008.08.137. PubMed: 18774777. 18774777

[30] Oppenheimer H, Gabay O, Meir H, Haze A, Kandel L et al. (2012) 75-kd sirtuin 1 blocks tumor necrosis factor alpha-mediated apoptosis in human osteoarthritic chondrocytes. Arthritis Rheum 64: 718-728. doi:10.1002/art.33407. PubMed: 21987377. 21987377 PMC3269551

[31] Kozako T, Aikawa A, Shoji T, Fujimoto T, Yoshimitsu M et al. (2012) High expression of the longevity gene product SIRT1 and apoptosis induction by sirtinol in adult T-cell leukemia cells. Int J Cancer 131: 2044-2055. doi:10.1002/ijc.27481. PubMed: 22322739. 22322739

[32] Firestein R, Blander G, Michan S, Oberdoerffer P, Ogino S et al. (2008) The SIRT1 deacetylase suppresses intestinal tumorigenesis and colon cancer growth. PLOS ONE 3: e2020. doi:10.1371/journal.pone.0002020. PubMed: 18414679. 18414679 PMC2289879

[33] Wu M, Wei W, Xiao X, Guo J, Xie X et al. (2012) Expression of SIRT1 is associated with lymph node metastasis and poor prognosis in both operable triple-negative and non-triple-negative breast cancer. Med Oncol 29: 3240-3249. doi:10.1007/s12032-012-0260-6. PubMed: 22661383. 22661383

[34] Wang RH, Sengupta K, Li C, Kim HS, Cao L et al. (2008) Impaired DNA damage response, genome instability, and tumorigenesis in SIRT1 mutant mice. Cancer Cell 14: 312-323. doi:10.1016/j.ccr.2008.09.001. PubMed: 18835033. 18835033 PMC2643030

[35] Fu M, Liu M, Sauve AA, Jiao X, Zhang X et al. (2006) Hormonal control of androgen receptor function through SIRT1. Mol Cell Biol 26: 8122-8135. doi:10.1128/MCB.00289-06. PubMed: 16923962. 16923962 PMC1636736

[36] Xie Y, Zhang J, Xu Y, Shao C (2012) SirT1 confers hypoxia-induced radioresistance via the modulation of c-Myc stabilization on hepatoma cells. J Radiat Res 53: 44-50. doi:10.1269/jrr.11062. PubMed: 22302044. 22302044

[37] Chang CJ, Hsu CC, Yung MC, Chen KY, Tzao C et al. (2009) Enhanced radiosensitivity and radiation-induced apoptosis in glioma CD133-positive cells by knockdown of SirT1 expression. Biochem Biophys Res Commun 380: 236-242. doi:10.1016/j.bbrc.2009.01.040. PubMed: 19166820. 19166820

[38] Matsushita N, Takami Y, Kimura M, Tachiiri S, Ishiai M et al. (2005) Role of NAD-dependent deacetylases SIRT1 and SIRT2 in radiation and cisplatin-induced cell death in vertebrate cells. Genes Cells 10: 321-332. doi:10.1111/j.1365-2443.2005.00836.x. PubMed: 15773895. 15773895

[39] Brooks CL, Gu W (2009) Anti-aging protein SIRT1: a role in cervical cancer? Aging (Albany NY) 1: 278-280. PubMed: 20157516. 20157516 10.18632/aging.100031 PMC2806011

[40] Brooks CL, Gu W (2009) How does SIRT1 affect metabolism, senescence and cancer? Nat Rev Cancer 9: 123-128. doi:10.1038/nrc2562. PubMed: 19132007. 19132007 PMC2857763

[41] Lovaas JD, Zhu L, Chiao CY, Byles V, Faller DV et al. (2013) SIRT1 enhances matrix metalloproteinase-2 expression and tumor cell invasion in prostate cancer cells. Prostate 73: 522-530. doi:10.1002/pros.22592. PubMed: 23038275. 23038275 PMC3839321

